# Perspectives of PDE inhibitor on treating idiopathic pulmonary fibrosis

**DOI:** 10.3389/fphar.2023.1111393

**Published:** 2023-02-14

**Authors:** Xudan Yang, Zhihao Xu, Songhua Hu, Juan Shen

**Affiliations:** Department of Respiratory and Critical Care Medicine, The Fourth Affiliated Hospital, School of Medicine, Zhejiang University, Yiwu, China

**Keywords:** pulmonary fibrosis, cAMP, cGMP, PDE inhibitor, anti-fibrosis, senescence

## Abstract

Idiopathic pulmonary fibrosis (IPF) is a chronic, progressive interstitial lung disease (ILD) without an identifiable cause. If not treated after diagnosis, the average life expectancy is 3–5 years. Currently approved drugs for the treatment of IPF are Pirfenidone and Nintedanib, as antifibrotic drugs, which can reduce the decline rate of forced vital capacity (FVC) and reduce the risk of acute exacerbation of IPF. However these drugs can not relieve the symptoms associated with IPF, nor improve the overall survival rate of IPF patients. We need to develop new, safe and effective drugs to treat pulmonary fibrosis. Previous studies have shown that cyclic nucleotides participate in the pathway and play an essential role in the process of pulmonary fibrosis. Phosphodiesterase (PDEs) is involved in cyclic nucleotide metabolism, so PDE inhibitors are candidates for pulmonary fibrosis. This paper reviews the research progress of PDE inhibitors related to pulmonary fibrosis, so as to provide ideas for the development of anti-pulmonary fibrosis drugs.

## 1 Introduction

IPF is a chronic, progressive age-related interstitial lung disease (ILD) of unknown etiology. If not treated after diagnosis, the average life expectancy is 3–5 years (King et al., 2011; Richeldi et al., 2017). Although the understanding of IPF has been significantly improved, the critical pathways of disease still need to be further explored. It is generally believed that environmental stressors and genetic susceptibility are the key factors that activate and promote the development of pulmonary fibrosis (Maher et al., 2007; Margaritopoulos et al., 2012). Genetic, environmental factors (smoking, dust, etc.), infection (EB virus, cytomegalovirus, herpesvirus), aging and other aspects interact to initiate continuous micro-damage of alveolar epithelial cells (Stewart et al., 1999; Lok et al., 2001; Tang et al., 2003; Maher et al., 2007; Richeldi et al., 2017). Alveolar macrophages recognize epithelial injury and amplify inflammatory response, secrete transforming growth factor-β (TGF-β),IL-10, platelet-derived growth factor (PDGF), and other cytokines, recruit fibroblasts to the injured site, transform fibroblasts into myofibroblasts, stimulate alveolar epithelial cells to undergo epithelial-mesenchymal transformation (EMT), and myofibroblasts secrete extracellular matrix components (ECM). It eventually leads to the occurrence, development and maintenance of pulmonary fibrosis (Craig et al., 2015; Pardali et al., 2017; Sgalla et al., 2018; Spagnolo et al., 2018). Many cell types and signaling pathways are involved in disease pathogenesis. The process of pulmonary fibrosis involves epithelial repair disorders, cell senescence, and immune response disorders. Because redundant cell types, growth factors, and fibrosis pathways are involved in the pathogenesis of the disease, there is still a lack of effective treatment for the progressive stage of IPF (Spagnolo et al., 2018).

Pirfenidone and Nintedanib, the latest two antifibrotic drugs, can significantly reduce the decline rate of forced vital capacity (FVC) within 1 year in IPF patients with mild and moderate lung function impairment. Moreover, it can reduce the risk of acute exacerbation in IPF patients with mild and moderate lung function impairment. Nintedanib is a small molecular tyrosine kinase inhibitor, which can inhibit platelet-derived growth factor receptor (PDGFR), fibroblast growth factor receptor (FGFR) and vascular endothelial growth factor receptor (VEGFR) on the cell surface. It competitively binds to adenosine triphosphate (ATP) binding sites on these intracellular receptor kinase domains, block intracellular signal transduction and inhibit fibroblast proliferation, migration, and transformation (Richeldi et al., 2017). Pirfenidone is a broad-spectrum anti-fibrotic drug with anti-inflammatory and anti-oxidant effects. Its mechanism is not completely clear (Spagnolo et al., 2020). However, the clinical studies had found that neither these two drugs alleviated the IPF-related symptoms nor improved the overall survival rate of IPF patients (Noble et al., 2011; King et al., 2014; Costabel et al., 2016). Furthermore, although Nintedanib and pirfenidone have good safety in clinical trials, there are still a few patients with adverse reactions, including nausea and vomiting, skin photosensitization, dizziness, and liver function damage (Richeldi et al., 2017). Therefore, we hope to explore more effective drugs for IPF. As with other fibrotic diseases, anti-fibrosis therapy focuses on avoiding tissue damage and eliminating remodeling of tissue parenchyma and function decline resulting from ECM deposition. Accumulating evidence suggests that cyclic nucleotides are involved in the regulation of pulmonary fibrosis.

Cyclic nucleotides cAMP and cGMP are typical second messengers. In the classical paradigm, in response to extracellular stimuli, cAMP or cGMP are respectively synthesized by adenylate cyclase (AC) or guanylate cyclase (GC) located on the plasma membrane, and then spread throughout the cell, where they interact with the specific effector proteins that regulate cell function, such as cell proliferation and differentiation, inflammation, apoptosis and metabolic pathways (Bolger, 2021). Phosphodiesterase (PDEs) is involved in cyclic nucleotide metabolism. So exploring the role of cyclic nucleotides and PDEs in the development of pulmonary fibrosis is helpful for developing anti-fibrosis drugs.

## 2 Mechanism of cyclic nucleotides regulating fibrosis

cAMP is an essential regulator of fibroblast function. The extracellular stimulators bind to the G protein-coupled receptor (GPCRs) on cell membrane, and adenylate cyclase (AC) responds to the activation of GPCRs to produce cAMP. cAMP enables the transmission of extracellular signals into the cell along defined and specific pathways within the network, allowing for signal regulation inside and outside the cell. This process, referred to as Compartmentalization, is a crucial aspect of cAMP signaling (Zuo et al., 2019a). cAMP participates in regulation mainly through four effectors: PKA (protein kinase A protein kinase, PKA), Epac (exchange protein activated by cAMP, Epac), cyclic nucleotide-gated (CNG) ion channels, and the Popeye domain-containing protein family (Zuo et al., 2019a). Furthermore, the cAMP/PKA pathway and the cAMP/Epac pathway have been reported the most in pulmonary fibrosis (Liu et al., 2004; Yokoyama et al., 2008; Insel et al., 2012).

The NO-GC-cGMP signaling pathway initiates with the catalytic conversion of arginine and molecular oxygen to NO and citrulline by nitric oxide synthase. After binding of lipophilic NO to sGC in the cytosol, sGC is fully activated and catalyzes the formation of the second messenger cGMP. cGMP can then bind to a variety of effectors to regulate cellular activity.

### 2.1 The cAMP/Epac pathway

**FIGURE 1:**
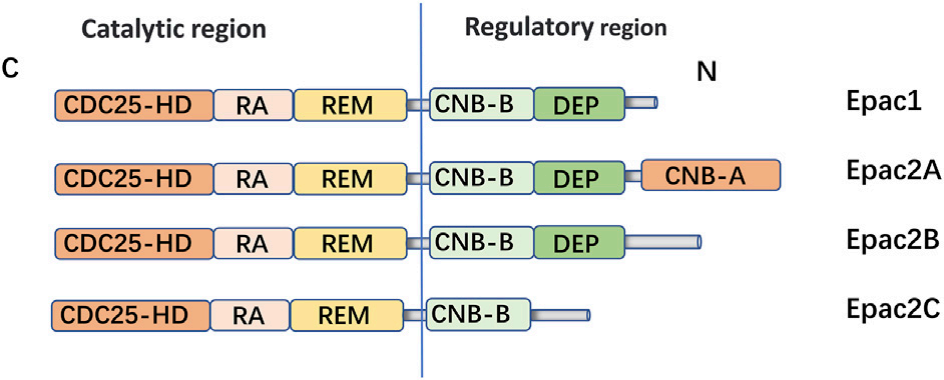
Domain architecture of EPAC isoforms: EPAC proteins are single polypeptide molecules which consist of an N-terminal regulatory region and a C-terminal catalytic region. The catalytic region at the C terminus is mainly composed of three structural and: Ras exchange motif (REM), Ras association (RA), CDC25 homology domain [also known as the guanine nucleotide exchange factor for Ras-like small GTPases (RasGEF) domain] responsible for nucleotide exchange activity. The two subtypes had different structures in the N-terminal regulatory region. The Epac2A regulation region contains two CAMP-binding domains, CNB-B and CNB-A. However, Epac1, Epac2A and Epac2B all have Disheveled/Egl-10/pleckstrin (DEP) domains, which are correlated with subcellular localization of Epac. Epac1 is widely expressed in human tissues, such as the hippocampus, thyroid, breast, and lung. EPAC2A is mainly expressed in the central nervous system, pituitary gland and adrenal gland.

**FIGURE 2:**
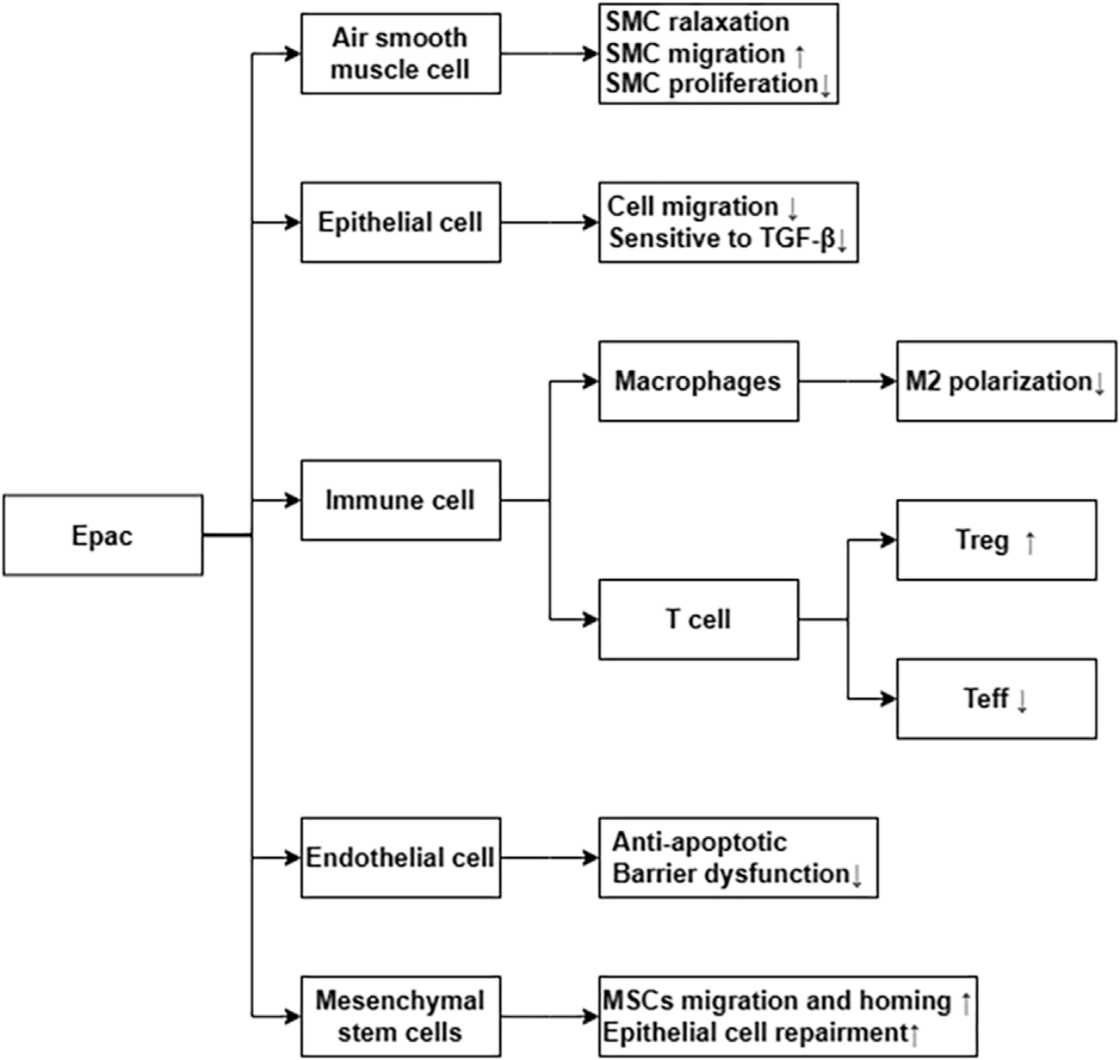
Epac *via* differential cellular pathways inhibit the process of IPF. ↑ means increase or upregulated; ↓ means decrease or downregulated.

Epac is widely found in lung, brain, and kidneyed. It participates in cAMP-mediated signal transduction by activating Ras-like small GTP enzyme Rap (Kawasaki et al., 1998). In cooperatione with PKA or alone, it undertakes numerous cAMP functions, such as regulating macrophage inflammation, epithelial cell adhesion, fibroblast proliferation, and differentiation. Epac consists of a regulatory region at its N-terminus and a catalytic region at the C-terminus. Based on the differences in the N-terminal regulatory region, Epac can be divided into two subtypes: Epac1 and Epac2 (Figure 1) (Niimura et al., 2009). By comparing pulmonary fibroblasts from normal patients to those from pulmonary fibrotic patients using Western blot, the study demonstrates that Epac1 is primarily expressed in the former group (Huang et al., 2008a). So, Epac1 may be responsible for the anti-fibrosis effect in the lung. Moreover, the lower cAMP concentration prioritizes the Epac pathway’s activation (Yokoyama et al., 2008). Based on these findings, Epac appears to be an attractive therapeutic target for the treatment of pulmonary fibrosis. Epac can regulate the proliferation, migration, and relaxation of airway smooth muscle cells (ASMC) in IPF, thereby correcting dysfunction and retarding the progression of IPF, which may be due to the reduction of RhoA activity by cAMP/Epac/Rap1 signaling (Roscioni et al., 2011; Zieba et al., 2011). Epac agonist promotes endothelial cells (ECs) survival by reducing the activities of pro-apoptotic caspases in a PI3K/Akt and MEK/ERK signalling-dependent manner (Gündüz et al., 2019). Besides, inhibition of MEK/ERK signaling enhances the stabilizing and protective effects of cAMP/Epac activation on endothelial cell barrier, indirectly inhibiting the progression of pulmonary fibrosis (Gündüz et al., 2019). TGF-β is an important profibrotic factor. In lung epithelial cells, EPAC is involved in the inhibition of transforming growth factor-β-dependent cell migration and adhesion, and endogenous TGFRI can form a complex with EPAC1 (Conrotto et al., 2007). In immune cells, Epac also can reverse the polarization of macrophages to pro-fibrotic M2, the mechanism of which remains to be explored (Hartopo et al., 2013). The antifibrotic effect of Epac may be multifaceted. Therefore, the anti-fibrosis mechanism is not well explained (Yokoyama et al., 2008). T cells also play an important role in the development of pulmonary fibrosis. In the early stage of pulmonary fibrosis, the major effector target T cells are regulatory T cells (Tregs). Treg are involved in early pulmonary fibrosis by secreting pro-fibrotic factors such as TGF-β, PDGF (Hou et al., 2017). However, as a homeostatic regulator of the immune response, Tregs can also mediate upstream inflammatory events and indirectly reduce the development of fibrosis by suppressing inflammation and T helper cell responses (Wilson and Wynn, 2009). Therefore, Tregs may have a different role in the process of pulmonary fibrosis at each stage. Epac1 can boosts Treg-mediated suppression effector T-cells (Teffs) while sensitizing Teffs to suppression (Almahariq et al., 2015). So, activation of Treg cells and regulating cAMP/EPAC in T cells may become a new strategy for the prevention and treatment of IPF. Mesenchymal Stem Cells (MSCs) is also involved in pulmonary fibrosis, but the effects are multifaceted. On the one hand, MSCs migrate to sites of lung injury to renew injured epithelial cells (Toonkel et al., 2013). On the other hand, the migration and adhesion of mesenchymal stem cells contribute to their differentiation into myofibroblasts and aggravate pulmonary fibrosis (El Agha et al., 2017). However, MSCs has been suggested as a therapy for the treatment of IPF (Toonkel et al., 2013). cAMP/Epac/Rap1 can promote the homing and migration of MSCs by enhancing stromal cell derived factor 1 (SDF-1), thereby enabling the repair of lung epithelial cells (Toonkel et al., 2013). A summary of the above cell types’ correlation with EPAC and IPF is shown in Figure 2.

### 2.2 The cAMP/PKA pathway

**FIGURE 3:**
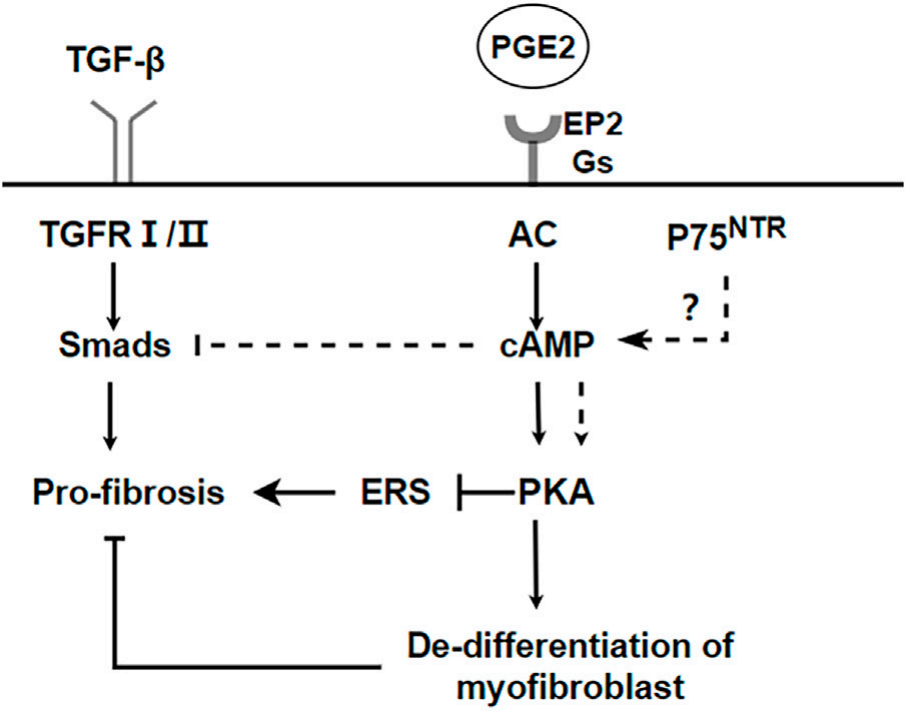
PKA pathways inhibit the process of IPF. cAMP/PKA can inhibit endoplasmic reticulum stress and promote dedifferentiation of myofibroblasts to realize anti-fibrosis. p75^NTR^ regulates tissue fibrosis through inhibition of plasminogen activation *via* a PDE4/cAMP/PKA pathway. But it needs to be further verified in pulmonary fibrosis.

PKA is also involved in the regulation of fibrosis. Endoplasmic reticulum stress (ER stress) contributes to the apoptosis of type II alveolar epithelial cells (AECs), which are involved in the process of pulmonary fibrosis (Kropski and Blackwell, 2018; Borok et al., 2020). ER stress stimulates NLRP3 inflammasome activation and promotes the process of lung fibrosis (Stout-Delgado et al., 2016). However, cAMP/PKA is a negative feedback regulator of ER stress-induced NLRP3 inflammasome activation, decreasing ACEⅡ pyroptosis (Hong et al., 2022). Prostaglandin (PG) E2 is a metabolite of arachidonic acid, mainly produced by alveolar epithelial cells and lung fibroblasts (Wilborn et al., 1995). Although it is a proinflammatory factor, it is essential in maintaining lung homeostasis. In the lung, PGE2 inhibits cell migration, proliferation, collagen accumulation, and differentiation into myofibroblasts (Elias et al., 1985; Wilborn et al., 1995; Kohyama et al., 2001). Therefore, PGE2 is considered to be a protective factor in pulmonary fibrosis. The downstream signal transduction of PGE2 is realized by binding to G protein coupled receptor EP1-EP4. However, Gαs-coupled E prostanoid (EP) 2 receptor will lead to an increaseing in cAMP (Huang et al., 2007). Therefore, the antifibrotic effect of PGE2 may be realized by the downstream effect mediated by cAMP.PGE2/Epac1/Rap pathway activation inhibites fibroblast proliferation, whereas PGE2/PKA activation inhibites collagen expression (Huang et al., 2008b). Futhermore, PGE2 can induce de-differentiation of human pulmonary myofibroblasts through cAMP/PKA pathway (Fortier et al., 2021). p75 neurotrophin receptor (p75^NTR^), a TNF receptor superfamily member upregulated after tissue injury, is involved in the regulation of proteolytic activity and fibrin degradation (Sachs et al., 2007). In neuronal tissues, p75^NTR^ regulates tissue fibrosis through inhibition of plasminogen activation *via* a PDE4/cAMP/PKA pathway. However, p75^NTR^ is also expressed in lung inflammation (Renz et al., 2004). Therefore, the p75^NTR^/PDE4/cAMP/PKA pathway it is a potential target for the study of pulmonary fibrosis. Respectively, activating the EPAC and PKA pathways, with the cAMP analogs 8-Me-cAMP and N6-cAMP, can reduce the sensitivity of fibroblasts to TGF-β and the production of myofibroblasts and extracellular matrix (ECM) (Insel et al., 2012). Recently, it has been reported that a new pan-PDE inhibitor shows anti-fibrosis effect in lung tissue by inhibiting the TGF- β signal pathway and activating the cAMP/PKA pathway (Wójcik-Pszczoła et al., 2020). A summary of the above the PKA and IPF is shown in Figure 3.

### 2.3 NO/SGC-cGMP pathway

cGMP can regulate heart, kidney, and liver fibrosis through the NO/SGC-cGMP pathway (Schinner et al., 2015; Sandner et al., 2017; Flores-Costa et al., 2018; Das et al., 2020). According previous studies, myofibroblasts responsible for lung damage in other ways besides the activity of contraction. The contractile force provides a feedforward mechanism, that maintains the differentiation of myofibroblasts in lung fibrosis. This is accomplished by converting mechanical stimuli into biochemical signals, which drive fibrosis progression (Desmoulière et al., 2005; Hinz, 2007; Wipff et al., 2007; Wynn and Ramalingam, 2012). Relaxin is a peptide hormone that regulates the production and degradation of collagen, and it is responsible for mediating the antifibrotic effects of collagen. Relaxin regulates myosin light chain (MLC20) dephosphorylation and lung myofibroblast contraction through the inactivation of RhoA/Rho-associated protein kinase by a nitric oxide/cGMP/protein kinase G (PKG)—dependent mechanism (Huang et al., 2011). Under conditions of high and persistent guanylyl cyclase activation, the activation of downstream cGMP can also reduce the differentiation of myofibroblasts induced by TGF-β (Dunkern et al., 2007).

**FIGURE 4:**
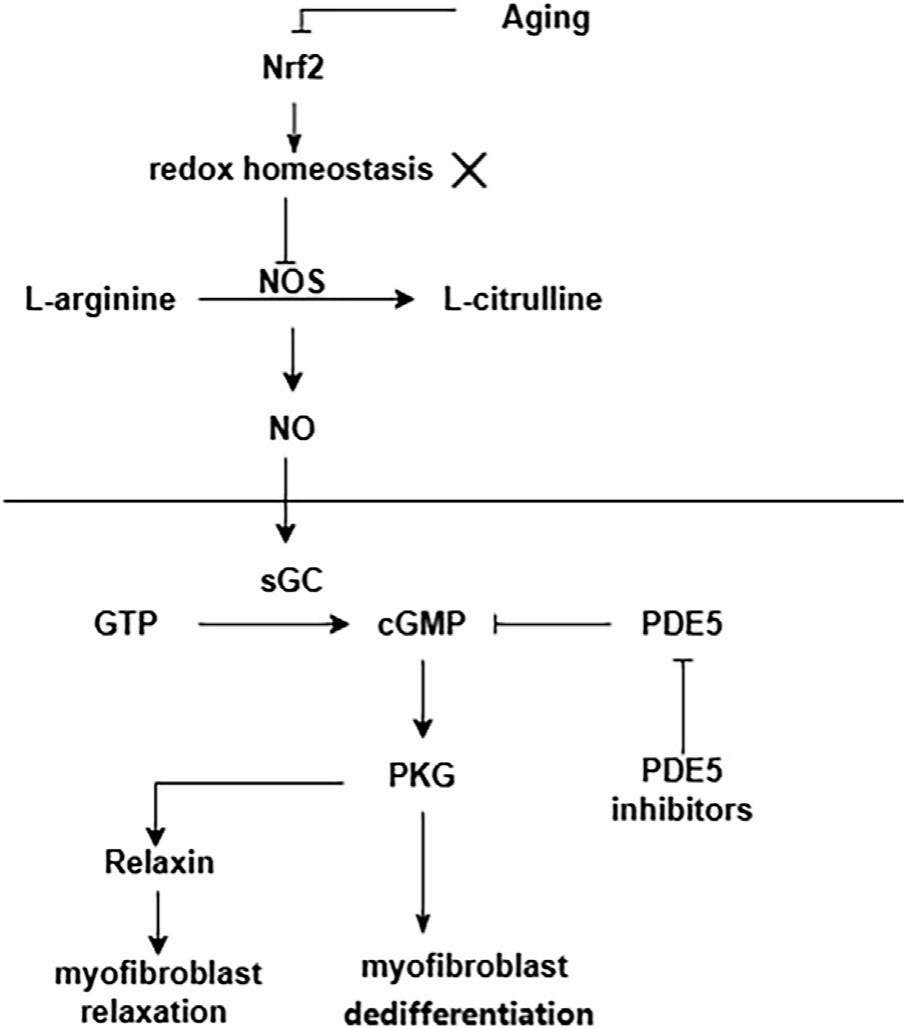
NO/sGC-cGMP pathway in pulmonary fibrosis. Aging leads to a decrease in the antioxidant capacity of Nrf2. This pro-oxidant shift results in NOS decoupling and a concurrent decrease in NO signaling and PKG activity.

Senescence is an important factor in the process of pulmonary fibrosis (Schafer et al., 2017; Justice et al., 2019; Otoupalova et al., 2020; Spagnolo et al., 2021a; Yao et al., 2021). Senescent alveolar epithelial cells and lung fibroblasts contribute to pulmonary fibrosis by secreting senescence-associated secretory phenotype (SASP) (Lin and Xu, 2020). Aging is accompanied by the increased oxidative stress and the accumulation of advanced glycation end products (AGEs), both of which are associated with the development of fibrosis (Richter and Kietzmann, 2016). One of the most important regulators of antioxidant genes is NFE2-related factor 2 (Nrf2). The antioxidant capacity of Nrf2 is reduced in the lung fibroblasts of aged mice, which results in a dynamic imbalance of cell redox homeostasis (Hecker et al., 2014). This pro-oxidant shift results in NO synthase (NOS) decoupling and a concurrent decrease in NO signaling and PKG activity (Sampson et al., 2012). Furthermore, in a variety of fibrotic diseases and also during the natural course of aging, NO/cGMP production is low (Sandner et al., 2017). Enhancement of NO/cGMP signaling by sGC stimulators or sGC activators ameliorates the development of fibrosis in various organs and tissues (Sandner et al., 2017). A summary of the above the NO/sGC-cGMP pathway shown in Figure 4.

### 2.4 Structure and subtype of PDE

PDEs work by hydrolyzing the phosphodiester bonds of the cyclic nucleotides, cyclic adenosine 3′,5′-monophosphate (cAMP) and cyclic guanosine 3′,5′-monophosphate (cGMP), which terminates the downstream signalling of this second messenger. It is subdivided into eleven subtypes based on its diverse structure. Through selective splicing or transcriptional modification of mRNA, these genes produce nearly one hundred PDE isozymes (Azevedo et al., 2014). The structures that make up the PDE superfamily are related but functionally distinct. These differences include tissue distribution, cellular function, primary structure, affinity for cAMP and cGMP, catalytic properties, and responses to specific activators, inhibitors, and effectors and their regulatory mechanisms. PDE4, PDE7, and PDE8 are PDEs that specifically degrade cAMP, while some PDEs specifically degrade cGMP (PDE5, PDE6, and PDE9) (Maurice et al., 2014). Most cells contain more than one PDE family member but in varying amounts, proportions, and subcellular locations. Although PDEs exhibit a broad tissue distribution, some cells are relatively enriched in specific PDEs (Table 1).

**TABLE 1:** PDE family and Tissue expression.

PDE family	Tissue expression	Disease
PDE1	Significant in cardiac and vascular myocytes, central and peripheral neurons, lymphoid (T and B cells) and myeloid cells Conti and Beavo (2007), Francis et al. (2011), and Keravis and Lugnier (2012)	Alzheimer’s disease Cardiovascular disease Le et al. (2022).
PDE2	In the brain, myocytes, liver, adrenal cortex,T cell endothelium and platelets Conti and Beavo (2007), Francis et al. (2011), Keravis and Lugnier (2012), and Michie et al. (1996)	Cardiovascular Diseases Sadek et al. (2020). Cognitive Impairment Abdel-Magid (2017).
PDE3	Cardiac and vascular myocytes, brain, liver, adipose tissues, airway cells Conti and Beavo (2007), Francis et al. (2011), Keravis and Lugnier (2012), and Beute et al. (2018)	Allergic airway inflammation Spagnolo et al. (2018). Age-Related Cognitive Impairment Yanai and Endo (2019) Cardiomyopathy Movsesian (2003).
PDE4	Broad; significant in cells of the cardiovascular, neural, immune and inflammatory systems Conti and Beavo (2007), Francis et al. (2011), and Keravis and Lugnier (2012)	Airway inflammatory diseases: COPD, asthma Phillips (2020). Alzheimer’s disease Gurney et al. (2015). Inflammatory bowel disease Li et al. (2022).
PDE5	vascular myocytes, lung, brain, platelets, kidney, gastrointestinal tissues and penis Conti and Beavo (2007), Francis et al. (2011), and Keravis and Lugnier (2012)	Erectile dysfunction Greco et al. (2006). Pulmonary hypertension Barnes et al. (2019). Neurological disorders: Alzheimer’s disease Zuccarello et al. (2020), Primary Hippocampal Neuronal Death Xu et al. (2020). Obesity and metabolic syndrome Armani et al. (2011).
PDE6	Photoreceptors and pineal gland Conti and Beavo (2007), Francis et al. (2011), and Keravis and Lugnier (2012)	Retinal diseases Gopalakrishna et al. (2017) and Wang et al. (2018)
PDE7	Spleen, brain, lung and kidney and lymphoid Conti and Beavo (2007), Francis et al. (2011), and Keravis and Lugnier (2012)	Autoimmune Disorders:, autoimmune Hepatitis Świerczek et al. (2021) Central nervous system diseases Chen et al. (2021): Parkinson’s disease (PD), Alzheimer’s disease (AD), multiple sclerosis (MS),
PDE8	Thyroid, airway smooth muscle, T cell Dong et al. (2006), Conti and Beavo (2007), Francis et al. (2011), Keravis and Lugnier (2012), and Basole et al. (2022)	Inflammatory Dong et al. (2006)
PDE9	Spleen, brain, cardiac, intestinal cells, lower urinary tract, Bender and Beavo (2006), Conti and Beavo (2007), Francis et al. (2011), Keravis and Lugnier (2012), Nagasaki et al. (2012), and Dunkerly-Eyring and Kass (2020)	Obesity and cardiometabolic syndrome Mishra et al. (2021) Alzheimer’s Disease Rabal et al. (2019)
PDE10	Brain, pancreatic Conti and Beavo (2007), Francis et al. (2011), and Keravis and Lugnier (2012)	Neurological disorders: Huntington’s Disease Models Beaumont et al. (2016), Mental illness: schizophrenia Abdel-Magid (2013)
PDE11	Prostate, testes and salivary and pituitary gland Conti and Beavo (2007), Francis et al. (2011), and Keravis and Lugnier (2012)	Depressive disorder Bollen and Prickaerts (2012)

PDEs contain two functional regions, regulatory and catalytic. The catalytic region determines the specificity to the substrate or inhibitor. The amino-terminal regulatory regions of PDEs are highly heterogeneous, reflecting the different cofactors of PDE family members (Maurice et al., 2014). A-kinase anchoring proteins (AKAPs) are anchoring proteins that anchor PKA to specific subcellular sites. AKAPs, PDEs, together, keep cAMP signalling specific and physically compartmentalised. As PDEs are the only route to cyclic nucleotides degradation, the specificity of the temporal and spatial distribution of PDEs ensures the viability of signal transduction. Without PDE-dependent control of local cAMP levels, intracellular cAMP, cGMP would be distributed indiscriminately. As a result, signalling specificity would be lost, as all subpopulations of PKA present in the cell would be activated (Brescia and Zaccolo, 2016). In this scenario, manipulation cyclic nucleotides of levels by specific pharmacological inhibition of individual PDE families is an effective treatment.

## 3 Studies of different classes of PDE inhibitors in pulmonary fibrosis

### 3.1 PDE4 inhibitor

PDE4 is a cAMP-specific PDE with relatively high expression levels in cells that regulate immune inflammatory responses and tissue remodeling (Torphy, 1998; Maurice et al., 2014), including macrophage activation (Hertz et al., 2009; Li et al., 2018). Furthermore, primary alveolar A549 cells and human bronchial epithelial (HBE) cells highly express PDE4 (Mata et al., 2005; Oldenburger et al., 2012). The non-selective PDE4 inhibitor Roflusteride is approved for use in severe COPD and acute exacerbations due to its anti-inflammatory properties (Hatzelmann et al., 2010). Studies have shown that PDE4 inhibitors can inhibit the release of fibrogenic factors and alleviate pulmonary fibrosis in a mouse model induced by bleomycin (Cortijo et al., 2009; Udalov et al., 2010; Milara et al., 2015). In transgenic mice expressing diphtheria toxin receptor under the control of the mouse surfactant protein C promoter (a model of pulmonary fibrosis targeting type Ⅱalveolar epithelial injury), PDE4 inhibitor also downregulated plasma levels of selective chemokines, and significantly reduces lung fibrosis induced by targeted type II AEC injury (Sisson et al., 2018). *In vitro*, PDE4 inhibitors inhibites FN-induced aggregation and collagen synthesis of human fetal lung fibroblasts (HFL-1), downregulates the sensitivity of fibroblasts to TGF-β, and promotes the inhibition of fibroblast function by prostaglandin E2 (PGE2) in the presence of PDE4 (Udalov et al., 2010). By decreasing reactive oxygen species, and extracellular signal-regulated kinase phosphorylation, the PDE4 inhibitor Rolipram or PDE4 small interfering RNA effectively inhibits EMT changes in a Smad-independent manner in the human alveolar epithelial type II cell line A549 (Kolosionek et al., 2022). Therefore, PDE4 inhibitors are the potential drug for IPF.

Still, the probability of side effects of non-selective PDE4 inhibitors, such as diarrhea, headache, nausea, and vomiting, makes the use of PDE4 inhibitors in patients limited (Spina, 2008; Maurice et al., 2014). These inhibitors have unfavorable side effects because they inhibit not just one PDE but an entire family of PDEs. It is known as the off-target effect. A coin has two sides, so as the off-target effects. On the one hand, it may increase drug toxicity and cause severe adverse reactions. However, acting with multiple targets may produce synergistic effects that amplify drug effects. For example, methylxanthine theophylline is a purine derivative, and it inhibits almost all types of PDEs. Theophylline can be used in asthma to dilate the bronchi by inhibiting PDE. And its anti-inflammatory actions -- which are mediated *via* inhibition of the nuclear translocation of nuclear factor-κb may be attributed to both PDE inhibition and increased cAMP signaling (Minguet et al., 2005). However, the therapeutic window of theophylline is narrow and toxic symptoms are easy to occur (Jacobs et al., 1976).

According to the difference between the transcriptional initiation site and selective mRNA splice site, PDE4 can be divided into four subtypes of PDE4A-D. In human primary lung fibroblasts (NHLF), PDE4A, B, and D are mainly expressed, while PDE4C is slightly or not present. Knockdown of PDE4B by SiRNA interference resulted in the most significant decrease in overall PDE4 enzyme activity, followed by PDE4A and PDE4D. PDE4B and 4D knockdown can inhibit the expression of α-SMA in TGF-βinduced pulmonary fibroblasts, in which the inhibition of PDE4B knockdown is the most effective, and the effect is similar to the non-selective PDE4 inhibitors (Selige et al., 2011). The adverse effects of PDE4 inhibitors appear to be related to the inhibition of PDE4D (Giembycz, 2001; Maurice et al., 2014). Therefore, PDE4B inhibitors seem to be ideal selective antifibrotic drugs.

BI101550, a PDE4 inhibitor with a high affinity to PDE4B, has anti-inflammatory and anti-fibrosis effects. *In vitro*, BI 1015550 inhibits lipopolysaccharide (LPS) induced TNF-α and phytohemagglutinin induced interleukin-2 synthesis in human peripheral blood mononuclear cells, as well as LPS-induced TNF-α synthesis in human and rat whole blood (Herrmann et al., 2022). In two mouse models of pulmonary fibrosis induced by bleomycin and silica, compared with a low dose (2.5 mg/kg), the higher BI1015550 (12.5 mg/kg b.i.d.) could improve the pulmonary function parameters of mice. High-dose BI1015550 could also significantly improve the content of dense fibrotic tissue in lung tissue. There is a synergistic effect between Nintedanib and BI1015550, which shifts the concentration-response curve to the left (Herrmann et al., 2022). Compared to roflumilast, BI 1015550 seems to be a safer option, and the male *Suncus murinus* is less likely to experience nausea and vomiting as a side effect (Herrmann et al., 2022). In a randomized, double-blind, placebo-controlled study involving 147 patients with IPF, the primary endpoint was the change from baseline in forced vital capacity (FVC) during 12 weeks of treatment with BI1015550 as monotherapy or in combination with antifibrotic background therapy. The trial results showed that BI1015550 at a dose of 18 mg twice daily prevented a decline in lung function in patients with IPF, regardless of background antifibrotic therapy. However, at the same time, the safety of BI1015550 is also of concern, with the most common adverse event being gastrointestinal disease. A case of “suspected IPF exacerbation and suspected vasculitis” was also reported (Richeldi et al., 2022). The adverse reactions need to be further evaluated in subsequent clinical trials. Nevertheless, BI1015550 is currently leading the way in the research and development of new drugs for the treatment of IPF.

There are other types of PDE4 inhibitors reported at present. AA6216 is a novel PDE4 inhibitor. AA6216, also ameliorated pulmonary fibrosis in mice by inhibiting TGF-β release from macrophages. However, in contrast to other PDE4 inhibitors, AA6216 possesses a more potent inhibitory effect with lower risk (Matsuhira et al., 2020). A novel PDE4 inhibitor was obtained by hit-to-lead optimization of natural mangoside based on structure, and its anti-pulmonary fibrosis effect was similar to that of pirfenidone. More importantly, it is safe and has fewer adverse reactions (Huang et al., 2021).

### 3.2 PDE5 inhibitor

PDE5 hydrolyzes cGMP exclusively. The pharmacological effect of sildenafil, a representative drug of the PDE5 inhibitor, is to increase the intracellular cGMP level by inhibiting cGMP degradation. And then the NO/sGC-cGMP pathway, is used to upregulate potassium channels, inhibit calcium channels, reduce intracellular calcium concentration, and dilate blood vessels. Its clinical indications include pulmonary hypertension (PH) and erectile dysfunction (Giovannoni et al., 2010). Currently, there are many studies on PDE5 inhibitors in pulmonary fibrosis. Due to the degeneration of lung structure that accompanies the progression of pulmonary fibrosis, pulmonary hypertension will eventually develop. In multiple randomized, controlled clinical trials, the addition of sildenafil in the context of antifibrotic agents has not been found to have a significant effect on all-cause mortality, hospitalization, or acute exacerbations (Kolb et al., 2018; Behr et al., 2021; Kang and Song, 2021). But, the efficacy evaluation of sildenafil in IPF is inconsistent (Collard et al., 2007). Since sildenafil improves gas exchange function in patients with severe pulmonary fibrosis by dilating pulmonary vessels, it may be effective in IPF. However, it is still debatable whether pulmonary vessel dilation can improve pulmonary gas exchange function in IPF (Sakao et al., 2019). IPF-related pulmonary arterial hypertension is distinct from idiopathic pulmonary arterial hypertension.

On the one hand, due to the destruction of the alveolar structure, the dysfunction of pulmonary gas exchange results in hypoxic pulmonary vasoconstriction (HPV) (Sylvester et al., 2012; Sakao et al., 2019). Early pulmonary vasoconstriction may be a compensatory mechanism during the development of pulmonary fibrosis. Pulmonary vasoconstriction can maintain the ventilation/perfusion ratio (V/Q) balance. However, continuous pulmonary vasoconstriction will lead to vascular remodeling, resulting in a vicious cycle, which should not be allowed to develop (Sakao et al., 2005; Sakao et al., 2006).

On the other hand, due to the destruction of the alveolar structure in IPF, the respiratory membrane is disordered and thickened, lung diffusion function is decreased, and dilated pulmonary blood vessels will further mismatch V/Q (Sakao et al., 2019). But is it feasible to use PDE5 inhibitors in the early stages of disease, when the structural damage of the lung is not apparent? Due to the insidious onset of IPF, non-specific clinical symptoms, and lack of diagnostic methods with high specificity for early IPF, most patients cannot be correctly diagnosed and treated at an early stage (Spagnolo et al., 2021b). By the time most patients are diagnosed with IPF, there is already apparent structural destruction of the lung. Consequently, there appears to be a lack of research on the potential benefits of initiating PDE5 inhibitor therapy at an early stage of IPF.

Additionally, sildenafil may contribute to pulmonary fibrosis through additional mechanisms. In a rat model of bleomycin-induced pulmonary fibrosis, sildenafil can reduce the oxidative stress level of lung tissue by inhibiting lipid peroxidation, the production and release of cytokines, and the aggregation of neutrophils, so as to achieve the therapeutic effect on pulmonary fibrosis (Yildirim et al., 2010). However, PDE5 inhibitors are not, according to the recommendations of international guidelines, appropriate treatment for IPF (Raghu et al., 2022).

### 3.3 Non-selective phosphodiesterase inhibitor

Pentoxifylline (PTX) is a methylxanthine derivative and non-selective phosphodiesterase inhibitor. Clinically, it is mainly used to improve peripheral circulation and relieve muscle pain caused by peripheral arterial diseases (Hood et al., 1996; Stevens et al., 2012). Previous studies have demonstrated that PTX can significantly inhibit the secretion of proinflammatory cytokines and the activation of NF-kB, thereby alleviating chronic inflammation (Speer et al., 2017). In RAW264.7 macrophages, the low dose of PTX (10 μg/mL) and the high dose of PTX (300 μg/mL) had different biological effects on cells. Low-dose PTX can reduce endoplasmic reticulum stress (ERS), fibrosis, angiogenesis, and chronic inflammation while promoting RAS/NF-kB signal transduction, proliferation, differentiation, and inflammation. It can also enhance Fas-mediated apoptosis. High doses of PTX, on the other hand, have the opposite effect by preventing RAS/NF-kB signal transduction, which prevents cell proliferation, inflammation, and fibrosis (Seo et al., 2022). It also suggests that PTX is a potential antifibrotic drug. PTX has an anti-fibrosis effect on radiation-induced pulmonary fibrosis by regulating the expression of PKA and PAI-1 (Lee et al., 2017; Wen et al., 2017). Our previous research found that, PTX could influence the expression of fibrosis-related genes in the mouse model of pulmonary fibrosis. In addition, we also found that the expression of senescence-associated secretory phenotype (SASP) decreased in the PTX group (Lin et al., 2022). Therefore, we speculated that PTX might may also alleviate pulmonary fibrosis through anti-aging. mTOR (mechanistic target of rapamycin) is a serine/threonine kinase involved in the integration of multiple metabolic and growth-promoting signals. Accumulating evidence indicates that mTOR activity is necessary for cell senescence (Liu and Sabatini, 2020). A decrease in mTOR activation was also observed after PTX treatment in human melanoma cells (Sharma et al., 2016). So we hypothesized that PTX may achieve its anti-fibrosis effect by affecting the mTOR pathway and changing the autophagy level of senescent cells. But that still needs to be tested. In other fibrosis models, including intestinal fibrosis, hypertrophic scar, and glomerulonephritis, PTX also has an anti-fibrosis effect (Boerma et al., 2008; Ng et al., 2009; Yang et al., 2019; Lee, 2022). The FDA approved PTX in 1984 for the treatment of arteritis. It is currently used to treat stroke because it improves circulation (Bath et al., 2000). So its safety in humans has been verified. “Drug repurposing” is a cost-effective option if the anti-fibrosis ability of PTX can be further developed.

### 3.4 Pan-PDE inhibitor

Because the antifibrotic effects of inhibitors of specific PDE subtypes are not clearly understood, and given the possible synergistic effects between different subtypes of PDE, the focus of some studies has shifted to dual PDE or pan-PDE inhibitors. Pan-PDE inhibitors represent compounds that can inhibit various isoforms within several PDE classes. Unlike simple PDE inhibitors, pan-PDE can inhibit individual PDE isoforms at the nano and/or micromolar level (Wójcik-Pszczoła et al., 2021). *In vitro* studies have shown that they have promising anti-inflammatory and antifibrotic activities and high inhibitory activity against a selection of PDEs. First, the PAN-PDE has significant inhibitory activity against multiple PDE isoforms, including PDE1, PDE3, PDE4, PDE5, PDE7, and PDE8, which are involved in airway remodeling and the development of pulmonary fibrosis (Wright et al., 1998; Fuhrmann et al., 1999). And next, considering the cAMP signaling compartmentalization during EMT, a different composition of individual isoforms within the cellular compartments cannot be ruled out (Zuo et al., 2019b; Wójcik-Pszczoła et al., 2022). *In vitro*, the pan-PDE inhibitors could inhibit the TGF-β-induced expression of several markers, including vimentin, fibronectin, collagen I, α-smooth muscle actin, N-cadherin, and snail-1 transcription factor in alveolar epithelial type II cells (Wójcik-Pszczoła et al., 2022).

## 4 Other novel anti-pulmonary fibrosis drugs

At present, there are other types of anti-pulmonary fibrosis drugs under development. The inflammasome NLR Family Pyrin Domain-Containing Protein 3 (NLRP3) is an important regulator of pulmonary inflammation and fibrosis (Colunga Biancatelli et al., 2022). NLRP3 promotes the development of pulmonary fibrosis mainly through the following aspects. Activated NLRP3 promotes fibrosis by producing IL-1 β and IL-18 (Colunga Biancatelli et al., 2022). NLRP3 mediated pyrolysis of csapase-1-dependent alveolar epithelial cells. NLRP3 induced pulmonary mesenchymal stem cells to differentiate into myofibroblasts (Ji et al., 2021). The activation of NLRP3 is increased in pulmonary fibrosis, and inhibition of NLRP3 can effectively delay the progression of pulmonary fibrosis, indicating that targeted NLRP3 may be a new choice for the treatment of pulmonary fibrosis. Although there are several NLRP3 inhibitors in existence, most of these drugs are still in the pre-clinical phase and there is a lack of validated data to confirm that they are indeed effective in pulmonary fibrosis. Furthermore, the indications for NLRP3 inhibitors are unclear. What clinical applications will show the best efficacy for NLRP3-targeting molecules? When, where, how is NLRP3 activated in human disease? To apply NLRP3 to pulmonary fibrosis, these questions need to be addressed.

GSDMD is a key effector of inflammasome signaling, because it controls pyroptosis and the resultant release of proinflammatory cellular contents. Given that GSDMD controls the release of IL-1β downstream of multiple inflammasomes, GSDMD is an attractive target for pulmonary fibrosis. Although, pyroptosis inhibitors will decrease the release of proinflammatory cell contents, they will not block the inflammasome-driven maturation of IL-1β or IL-18. Therefore, its anti-fibrosis effectiveness needs to be further confirmed. Heat-shock protein 90 (HSP) inhibitor, a new drug also developed from the NLRP3 inflammasome. As a multifunctional molecular chaperone, Hsp90 forms a complex with NLRP3 to protect NLRP3 from degrading. In response to stress stimuli, Hsp90 is released, and NLRP3 can be activated to promote inflammation. Hsp90 inhibition block the activation of the NLRP3 inflammasome. Inflammasome blockers show enormous promise as a new generation of anti-inflammatory drugs. However, these treatments are not mature at present, and there is still a long way to go before the real clinical application. Compared with PDEs inhibitors, the PDE inhibitors are more mature. Moreover, there are exact data to prove the effectiveness of PDEs inhibitors in pulmonary fibrosis.

Phosphatidylinositol 3-kinase (PI3K)/protein kinase B (PKB/AKT) signaling pathway plays an important role in IPF. TGF-βand PI3K/AKT promoted the formation of pulmonary fibrosis synergistically. PI3K/AKT can promote pulmonary fibrosis by regulating its downstreams such as mammalian target of rapamycin (mTOR), hypoxia inducible factor-1a (HIF-1a). Therefore, targeting PI3K/AKT has become a new strategy for the treatment of IPF (Conte et al., 2013; Nie et al., 2017). Some PI3K/AKT inhibitors has been investigated in clinical research. Reported treatment-related adverse event mainly include gastrointestinal effects. But for its effectiveness, there is a lack of data at present (Wang et al., 2022). However, PI3K/AKT still considered promising drug candidates for IPF treatment.

## 5 Discussion

Idiopathic pulmonary fibrosis is the most common type of idiopathic interstitial pneumonia. It is a progressive, irreversible and fatal disease. Its pathological mechanisms are complex and not well understood at present. Therefore, antifibrotic drugs are also limited. Although the current antifibrotic drugs, Pirfenidone and Nintedanib, have a certain therapeutic effect, they do not improve the prognosis of patients. Moreover, their current prices are relatively expensive. Therefore, we need to develop new antifibrotic drugs.

The cAMP signaling pathway is a relatively old signaling pathway, and scientists began to study it as early as 1953 (Zuo et al., 2019b). In the lung, cAMP, and cGMP mainly reduce the sensitivity of fibroblasts to pro-fibrotic factors and decrease the production of myofibroblasts. However, the underlying mechanisms need to be further explored. Our previous study shows that Pentoxifylline can inhibits pulmonary fibrosis by regulating cellular senescence. This suggests that we can study the possible mechanism of cyclic nucleotides against fibrosis from the point of view of aging. In recent years, an increasing number of studies have found that cAMP signalling also plays an important role in age-related cognitive deficits. So, Therefore, studying the role of cyclic nucleotides in pulmonary fibrosis from the perspective of aging may provide new ideas to understand the pathogenesis of pulmonary fibrosis further.

PDEs are involved in the metabolism of cyclic nucleotides, and their inhibitors can increase the intracellular concentration of cyclic nucleotides, thus exerting their anti-fibrotic effects. The “off-target” effect is a problem in the application of drugs, and it is a double-edged sword. On the one hand, the “off-target” result may have side effects on non-target sites, thus limiting the safe use of the drug. On the other hand, there may be synergistic effects between PDE isoforms, amplifying the drug’s therapeutic effects. A novel compound PDE inhibitor, Pan-PDE, is a good example. In contrast, PDE5 inhibitors, although some studies have shown some antifibrotic effects, whether IPF patients really benefit from them needs to be thoroughly evaluated.

Although there are many studies on PDEs inhibitors, most are still in animal experiments, and their effectiveness in humans needs further testing. But it is surprising that BI1O1550, a specific PDE4B inhibitor, has already started clinical trials. This is a big step forward in developing drugs to treat fibrosis.
